# The Gut Microbiota in Infants: Focus on *Bifidobacterium*

**DOI:** 10.3390/microorganisms11020537

**Published:** 2023-02-20

**Authors:** Gonzalo Solís, Miguel Gueimonde

**Affiliations:** 1 Pediatrics Service, Central University Hospital of Asturias (HUCA-SESPA), 33011 Oviedo, Asturias, Spain; 2 Instituto de Investigación Sanitaria del Principado de Asturias (ISPA), 33011 Oviedo, Asturias, Spain; 3 Instituto de Productos Lácteos de Asturias (IPLA), CSIC, 33300 Villaviciosa, Asturias, Spain

A long time has passed since the initial pioneering works were carried out on the composition of infant microbiota by Thedore Escherich (1857–1911) and Ernst Moro (1874–1951), and since the observations of Henry Tissier (1866–1916) which linked “Bacillus bifidus” to the health of babies. Nowadays, more than a century later, we are still studying the importance of infant microbiota and are trying to unravel the special interest of that so-called “Bacillus bifidus”, today known as *Bifidobacterium*, in this process. During the last two decades, the proliferation and increasing cost-effectiveness of next-generation sequencing techniques has allowed an unprecedented understanding on the composition and functions of microbiota along the human lifespan, from infancy to senescence, as well as their relation to general health. Several studies have underlined the critical importance of microbiota in early life stages for the development of host homeostasis and the later health of the individual. In this context, the genus *Bifidobacterium*, the dominant microorganism in the intestinal microbiota of healthy breastfed babies, is recognized as of paramount importance. These are aspects of current research interest since we are finally beginning to understand the full complexity of microbe–host interactions. However, our knowledge on the characteristics of the microbes and the factors affecting the development of specific bacterial populations, such as *Lactobacillus* and *Bifidobacterium*, remains limited. In this context, this Special Issue, entitled “The Gut microbiota in Infants: Focus on Bifidobacterium” covers different aspects of this research area, including original research articles comprising both in vitro and in vivo data, as well as reviews on the importance of bifidobacteria for infant health.

This Special Issue considers different aspects related to the topic. In their in vitro, study Harata and collaborators [1] assessed the adhesion ability of different species, including *Bifidobacterium bifidum*, to human intestinal mucus. The authors observed age-dependency, with some strains showing higher adherence to adult mucus, whereas others, such as *B. bifidum*, adhered better to mucin from infants. In vitro tests were also used for screening and selecting synbiotic combinations active against the pathogen *Staphylococcus aureus*, underlining the interest of combining fructans with *B. bifidum* [2]. The ability of some strains of bifidobacteria to ferment fructooligosaccharides and inulin has been repeatedly reported, making these bacteria an interesting approach for the development of synbiotic products. However, there is also an interest in including other microorganisms, such as *Lactobacillus*, in such symbiotic products. In this regard, in their article, Renye and coworkers [3] screened the ability of 86 strains of *Lactobacillus* to be grown in inulin and fructooligosaccharides, identifying strains suitable for the development of such products. Continuing with the study of the microbial interactions that may occur in the gut, the data obtained in a colonic simulation system, inlcuding *Bifidobacterium adolescentis* and the pathogen *Bacillus cereus*, were used by Evdokimova and coworkers [4] for modeling this interaction, allowing the prediction of the individual behavior of these microbes. 

Phenotypic and genotypic tests were used in the study of Duboux and colleagues [5] to decipher the diversity within the *Bifidobacterium longum* subp. *infantis* taxon, underlining existing differences in the utilization of human milk oligosaccharides (HMOs) and antibiotic resistance patterns. Moreover, the authors suggested a clonal relationship of some strains available nowadays with the first isolates from the species, dated back to the 1950’s. Additionally, taking advantage of comparative genomics, Diaz et al. [6] reported on the strain’s genomic divergence and the glucosyl hydrolases repertoire, related to the fermentation of HMOs, on the other main subspecies of *B. longum*: *Bifidobacterium longum* subsp. *longum*. Continuing with the interest in the interaction of the infant microbiota with HMOs, Nogacka and coworkers used an in vitro fecal culture model to assess the ability of the microbiota from infants to utilize 2’-Fucosyllactose [7]. Notably, the results indicated that not every baby’s microbiota are able to metabolize this compound and, therefore, perhaps not all babies would equally benefit from its inclusion in formulas and/or infant foods.

Two of the articles included in this Special Issue focus on the impact of perinatal factors in the development of the microbiota, with special emphasis on the bifidobacterial microbiota. Both of them remark the importance of intrapartum antimicrobial prophylaxis (IAP), the most common form of exposure to antibiotics in the perinatal period, being present in about 30% of deliveries. In the first study, Chen and colleagues [8] assessed the effect of IAP and c-section on the microbiota of newborns, observing that these two factors affect microbiota composition, including the levels of the genus *Bifidobacterium* and those of the subspecies *B. longum* subsp. *infantis*. In another study dealing with this topic, Saturio and coworkers used a bifidobacterial-ITS sequencing approach for characterizing the impact of IAP upon the development of bifidobacterial microbiota in babies during the first three months of life [9]. The authors observed quantitative and qualitative changes in different species of *Bifidobacterium*.

The interest in understanding early life microbiota and the factors determining their development is rooted in the demonstration of its association with general health. One of the aspects most extensively studied in this area is that of the infant microbiota and allergic diseases, which has also been the subject of different probiotic intervention studies. Climent at al. [10] reported the impact upon the microbiota of a probiotic product, containing a mixture of *Bifidobacterium* and *Lactobacillus* strains, previously found to improve atopic dermatitis symptoms in infants. Furthermore, the importance of bifidobacteria for infants’ health was reviewed in two articles included in this Special Issue. The first one reviewed the data on the primary colonization and the impact of bifidobacterial on host immunity, highlighting the key role that bifidobacterial metabolites have in this interaction [11]. Finally, the second review [12] focused on the importance of the genus *Bifidobacterium* for neonatal health and development and reviewed the evidence, from animal and human clinical studies, on the use of this microorganism as a probiotic for infants.

Overall this Special Issue covers the topic from a broad perspective and in a comprehensive way. The articles included in this issue provide new data and novel insights on the establishment of the neonatal bifidobacterial microbiota, the factors influencing it, and the importance of this process for the later health of the individual. The manuscripts also address to knowledge gaps and challenges that must be addressed to gain a full understanding of the importance of these interesting microorganisms for infant health. 

## References

[1] Harata G., Yoda K., Wang R., Miyazawa K., Sato M., He F., Endo A. (2021). Species- and Age/Generation-Dependent Adherence of *Bifidobacterium bifidum* to Human Intestinal Mucus *In Vitro*. Microorganisms.

[2] Evdokimova S.A., Nokhaeva V.S., Karetkin B.A., Guseva E.V., Khabibulina N.V., Kornienko M.A., Grosheva V.D., Menshutina N.V., Shakir I.V., Panfilov V.I. (2021). A Study on the Synbiotic Composition of *Bifidobacterium bifidum* and Fructans from *Arctium lappa* Roots and *Helianthus tuberosus* Tubers against *Staphylococcus aureus*. Microorganisms.

[3] Renye J.A., White A.K., Hotchkiss A.T. (2021). Identification of *Lactobacillus* Strains Capable of Fermenting Fructo-Oligosaccharides and Inulin. Microorganisms.

[4] Evdokimova S.A., Karetkin B.A., Guseva E.V., Gordienko M.G., Khabibulina N.V., Panfilov V.I., Menshutina N.V., Gradova N.B. (2022). A Study and Modeling of *Bifidobacterium* and *Bacillus* Coculture Continuous Fermentation under Distal Intestine Simulated Conditions. Microorganisms.

[5] Duboux S., Ngom-Bru C., De Bruyn F., Bogicevic B. (2022). Phylogenetic, Functional and Safety Features of 1950s *B. infantis* Strains. Microorganisms.

[6] Díaz R., Torres-Miranda A., Orellana G., Garrido D. (2021). Comparative Genomic Analysis of Novel *Bifidobacterium longum* subsp. *longum* Strains Reveals Functional Divergence in the Human Gut Microbiota. Microorganisms.

[7] Nogacka A.M., Arboleya S., Nikpoor N., Auger J., Salazar N., Cuesta I., Mantecón L., Solís G., Gueimonde M., Tompkins T.A. (2021). Influence of 2′-Fucosyllactose on the Microbiota Composition and Metabolic Activity of Fecal Cultures from Breastfed and Formula-Fed Infants at Two Months of Age. Microorganisms.

[8] Chen Y.Y., Zhao X., Moeder W., Tun H.M., Simons E., Mandhane P.J., Moraes T.J., Turvey S.E., Subbarao P., Scott J.A. (2021). Impact of Maternal Intrapartum Antibiotics, and Caesarean Section with and without Labour on *Bifidobacterium* and Other Infant Gut Microbiota. Microorganisms.

[9] Saturio S., Suárez M., Mancabelli L., Fernández N., Mantecón L., de los Reyes-Gavilán C.G., Ventura M., Gueimonde M., Arboleya S., Solís G. (2021). Effect of Intrapartum Antibiotics Prophylaxis on the Bifidobacterial Establishment within the Neonatal Gut. Microorganisms.

[10] Climent E., Martinez-Blanch J.F., Llobregat L., Ruzafa-Costas B., Carrión-Gutiérrez M.Á., Ramírez-Boscá A., Prieto-Merino D., Genovés S., Codoñer F.M., Ramón D. (2021). Changes in Gut Microbiota Correlates with Response to Treatment with Probiotics in Patients with Atopic Dermatitis. A Post Hoc Analysis of a Clinical Trial. Microorganisms.

[11] Stuivenberg G.A., Burton J.P., Bron P.A., Reid G. (2022). Why Are Bifidobacteria Important for Infants?. Microorganisms.

[12] Saturio S., Nogacka A.M., Alvarado-Jasso G.M., Salazar N., de los Reyes-Gavilán C.G., Gueimonde M., Arboleya S. (2021). Role of Bifidobacteria on Infant Health. Microorganisms.

